# Excessive Existence of Positively Charged Amino Acids Caused Off-Target Recognition in the Seed Region of *Clostridium butyricum* Argonaute

**DOI:** 10.3390/ijms26104738

**Published:** 2025-05-15

**Authors:** Wenzhuo Ma, Wenping Lyu, Lizhe Zhu

**Affiliations:** School of Medicine, Warshel Institute for Computational Biology, The Chinese University of Hong Kong—Shenzhen, Shenzhen 518172, China; wenzhuoma@link.cuhk.edu.cn (W.M.); lvwenping@cuhk.edu.cn (W.L.)

**Keywords:** off-target effect, path searching, Argonaute

## Abstract

*Clostridium butyricum* Argonaute (*Cb*Ago) can achieve DNA-guided DNA recognition and cleavage at physiological temperatures (~37 °C), making it a promising tool for gene editing. However, its significant off-target effects, particularly associated with the seed region (sites 2–8), pose challenges for precise gene therapy. This study focuses on enhancing the specificity of the seed region recognition to mitigate these off-target effects. We investigated the molecular recognition process between the *Cb*Ago-gDNA complex and the seed region of the target DNA using molecular dynamics simulations and automated path searching. Our findings reveal that positively charged residues located in an α-helix domain at the DNA–protein interface (R279, H285, K287, K288, K291, K298) facilitate rapid binding to the DNA phosphate backbone. Such interaction enhances the pre-formation of the DNA double helix, reducing the reliance on base complementarity during duplex pairing. Further simulations showed that alanine replacement of these positively charged residues led to significantly improved sequence specificity for the target DNA seed region. Collectively, these results offered critical insights into the origin of off-target recognition by *Cb*Ago in its seed region, shedding lights on its fidelity enhancement.

## 1. Introduction

**Figure 1:**
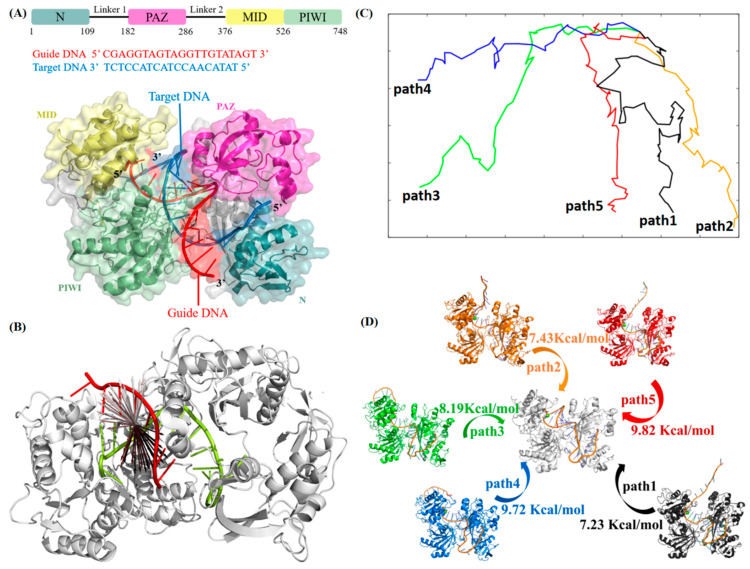
(**A**) The domain organization of *Cb*Ago and overall structure of the *Cb*Ago complex (PDB ID: 6QZK). Domains are rendered according to the color in the upper panel. (**B**) A schematic diagram showing different SMD directions, with the arrow starting at the center of mass of the 9-mer tDNA fragment (colored in red). gDNA and *Cb*Ago are indicated in green and gray, respectively. (**C**) 2D projection of five optimized LFEP transition paths by MDS. The larger the distance between any points on two paths, the lower their conformational similarity in 3D space. (**D**) Initial states of the five optimized paths and their highest free energy barrier of adjacent intermediate (IM) and transition state (TS) (dG_max_(IM-TS)) on free energy profile (Supplementary Figure S1).

Prokaryotic Argonaute (pAgo) proteins are enzymes that utilizes a guide nucleic acid strand (DNA or RNA), typically 20–22 nucleotide long, to recognize a complementary target nucleic acid strand so as to cleave the target in the microorganism. For the Argonaute of *Clostridium butyricum* (*Cb*Ago) [1], both the guide and target strand are single-stranded DNAs and cleavage of the target can already occur at moderate body temperatures (37 °C) [2], which presents *Cb*Ago as a potential gene editing tool that operates at physiological temperatures [3,4]. In 2019, Van der Oost et al. elucidated the crystal structure of *Cb*Ago through X-ray crystallography [1], providing detailed insights into the protein–DNA interface and the complete Watson–Crick base pairing spanning positions 2–16 of the guide DNA (gDNA) complex (Figure 1A). Specifically, *Cb*Ago recognizes target DNA (tDNA) through a preformed protein–DNA interface with gDNA in several sequential steps as follows [1]: (1) *Cb*Ago binds to the 5′ and 3′ ends of the gDNA separately via the MID and PAZ domains, forming a DNA silencing complex known as DNA Induced Silencing Complex (DISC); (2) the seed region of the guiding chain gDNA (positions 2–8, g2-g8) first pairs with its corresponding tDNA bases on the surface of *Cb*Ago, initiating base pairing; (3) further base pairing occurs at positions 9–12 (central region g9-g12) between the tDNA and the guiding chain; and (4) the 3′ end of gDNA dissociates from the PAZ domain of *Cb*Ago, completing base pairing at positions 13–16 (supplementary region, g13-g16) with tDNA. However, the reported *Cb*Ago tolerates mismatches in both the seed region and the central region of DNA [1,2,4], demonstrating a high off-target rate when employing *Cb*Ago for gene editing. This pronounced mismatch tolerance in both seed and central regions constitutes a principal factor underlying the unresolved challenge of off-target effects, which has precluded the successful implementation of *Cb*Ago-mediated genome editing applications. To facilitate its application in precision gene manipulation, obtaining high-fidelity *Cb*Ago mutants through strategic protein engineering seems an inevitable step.

The key to designing high-fidelity *Cb*Ago mutants lies in understanding the physical origin of its off-target effects, since blind mutagenesis would lead to a large body of experimental trials and a high failure rate. The mechanism underlying tDNA recognition—particularly how the binding of the seed region (g2–g8) permits mismatched base pairing in both in vivo and in vitro contexts—remains unclear. Notably, the dynamic conformational transitions of polymeric biomolecules, such as single-stranded DNA (ssDNA) [5,6,7], present significant challenges for characterization using conventional experimental techniques (e.g., X-ray crystallography [8,9] or static NMR spectroscopy [10,11]), especially when probing transient states at physiologically relevant timescales. Molecular simulations offer opportunities in capturing the temporal evolution of molecular structures and interactions [12,13], revealing the impact of mutations on the conformational dynamics [14,15,16].

To elucidate the recognition mechanism and energy landscape governing the interaction of the gDNA/*Cb*Ago complex with its cognate DNA substrates, we employed an integrated computational approach that combines automated path searching [17] and free energy profile calculations [18]. We identified five distinct low free energy pathways (LFEPs) for the conformational changes of the *Cb*Ago–gDNA–Tdna complex during the tDNA recognition at the seed region (g2–g8) of the gDNA. Additionally, we conducted simulations of a series of *Cb*Ago mutants, which validated the importance of the key amino acids we identified for rational design.

## 2. Results and Discussion

The five distinct LFEPs for the seed region recognition are all featured by low energy barriers ranging from 7 to 10 kcal/mol. In addition, all these LFEPs pointed to a facilitating role of a series of positively charged residues at the tDNA–protein interface (ARG279, HIS285, LYS287, LYS288, LYS291, LYS298) in the rapid coordination of these residues to the DNA phosphate backbone, regardless of the gDNA–tDNA sequence complementarity. Additional simulations showed that alanine substitutions of these residues led to a charge-attenuated *Cb*Ago variant (m*Cb*Ago) that exhibited higher free energy barriers, and, therefore, slower recognition of the mismatched tDNA than the on-target tDNA throughout the five LFEPs, and achieved seed fidelity enhancement.

### 2.1. Target DNA Recognition in the Seed Region of CbAgo

Target DNA recognition by the *Cb*Ago–guide DNA complex is believed to be initiated through thermodynamically driven base-pairing interactions between the guide positions g2–g8 and the corresponding target sequence [1] (Figure 1A). To investigate the molecular recognition of target DNA (tDNA) by the seed region (g2–g8) of guide DNA (gDNA), we conducted steered molecular dynamics [19] (SMD) simulations, starting from the base-paired state [1]. A complementary 9-mer single-stranded DNA (ssDNA) fragment was used to mimic the seed region of tDNA (Figure 1B). We performed a total of 8 independent SMD simulations with various pull directions to mechanically dissociate the ssDNA fragment from the gDNA/*Cb*Ago complex under constant velocity conditions (0.01 nm/ps). From these SMD trajectories, we selected five initial dissociation paths (indicated by arrows in Figure 1B) that exhibited the least number of collisions between the ssDNA and the *Cb*Ago/gDNA complex.

We then utilized our established path optimization algorithm, namely a travelling-salesman-based automated path searching method (TAPS) [17], to refine the five initial paths into the low free energy paths (LFEPs). The energy barriers for path1 to path5 are 7.23, 7.43, 8.19, 9.72, and 9.82 kcal/mol, respectively, indicating that these paths are closer approximations to the physical transition pathways. We found that the five optimized LFEPs can be well separated (Figure 1C) in a two-dimensional (2D) projection plot using multidimensional scaling [20] (MDS). This implies that multiple distinguishable transition pathways exist for the binding of tDNA onto the seed region (g2–g8) of gDNA (Figure 1C).

The free energy profile of each optimized transition path was estimated using umbrella sampling to examine the free energy barriers during the molecular recognition between the 9-mer tDNA fragment and the seed region of gDNA. We found that the molecular recognition of tDNA by the gDNA/*Cb*Ago complex underwent rugged free energy profiles with many traps (intermediates, IM) and barriers (transition states, TS) present across all five pathways (Supplementary Figure S1). The highest free energy barrier between adjacent IM and TS (dGmax(IM→TS)) on the five optimized transition paths ranges from 7 kcal/mol to 10 kcal/mol (Figure 1D). This is significantly smaller than the absolute value of the overall free energy difference (dG(initial→final)) of the 9-mer ssDNA fragment transitioning from the dissociated state to the base-paired state with the gDNA’s seed region. The ΔG(initial→final) of path1 to path5 are 26.27, 29.66, 28.51, 33.94, and 24.70 kcal/mol, respectively. This indicates that all five optimized transition pathways are favorable in terms of free energy changes. In other words, the molecular recognition between the 9-mer tDNA fragment and the seed region of gDNA is a multi-pathway process.

**Figure 2:**
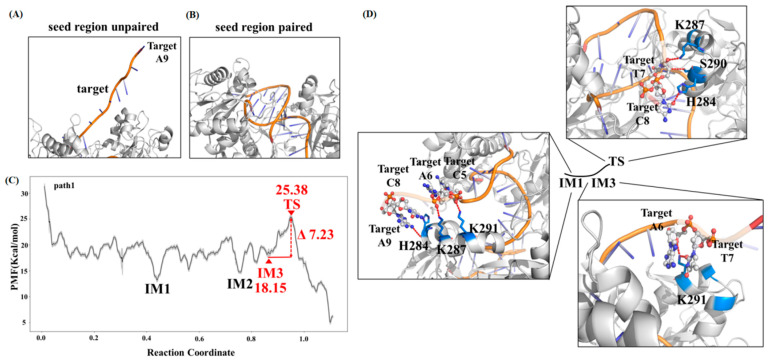
(**A**,**B**) The initial and final states of path1. (**C**) The free energy profile of conformation transition from the initial to final states of path1. The dG_max_(IM→TS) on this path is between IM3 and TS. (**D**) Interactions between tDNA and the gDNA/*Cb*Ago complex at IM1, IM3, and TS on path1.

To facilitate the following discussion, we named the five pathways as paths 1 to 5 according to the order of their highest free energy barriers dGmax(IM→TS) (Figure 1D). The dGmax(IM→TS) of path 1 is the lowest among the five paths, approximately 7.23 kcal/mol (Figure 1D). This path represents the optimal pathway for molecular recognition among the five obtained pathways. It delineates the transition process from an initial dissociated state (Figure 2A) to the final state (base-paired state, Figure 2B) through several free energy traps (IM) and barriers (TS), with the labeled IM1, IM2, IM3, and TS being particularly crucial (Figure 2C).

The initial structure of path 1 positions the 3′ end of the 9-mer tDNA fragment near the 5′ end of the guide DNA, while the 5′ end is distanced from the gDNA within the *Cb*Ago cavity (Figure 2A). Before the 9-mer tDNA fragment fully base pairs with the seed region of gDNA, it undergoes several free energy traps (IM1-3, Figure 2C) in sequence. Among these free energy traps, IM1 is the deepest, indicating that the conformational transition of the 9-mer tDNA is trapped at this state. However, only one base pair is formed between the tDNA and gDNA at the g2 position. Instead, LYS287 and LYS291 interact with the backbone of the tDNA at positions t5 and t6, respectively; HIS284 forms hydrogen bonds with the bases at positions t8 and t9 on the tDNA (IM1, Figure 2D). These findings suggest that the molecular recognition at IM1 is dominated not by base pairing, but by interactions between the tDNA and the protein’s polar residues. Interestingly, we find that the tDNA can slide on the positively charged surface of the protein during base pairing with gDNA: the interaction between LYS291 and the tDNA shifts to positions t6 and t7 after four base pairs are formed between the tDNA and gDNA at g2 to g5 positions sequentially (IM3, Figure 2D).

In the transition state of path 1, the backbones of the tDNA and gDNA essentially form a double helix-like structure, with only a slight remaining distance at the last two bases (t7–t8, TS, Figure 2D). Meanwhile, we observe that the backbone of the tDNA at the last two positions (t7 and t8) forms hydrogen bonds with LYS287 and HIS284, respectively. Additionally, SER290 interacts with the t7 position of the tDNA. However, none of these tDNA–protein interactions align with the direction of the tDNA’s movement towards the gDNA, resulting in internal tension within the ternary complex. This rationalizes the observed free energy barrier at this state. Indeed, once these tDNA–protein interactions are disrupted, the remaining base pairs are rapidly replenished to reach the final state with the lowest free energy (Figure 2C).

Although different initial and intermediate states are sampled along the other four transition paths (Supplementary Figures S2–S5), similar tDNA–protein interactions are observed contributing to the free energy traps or barriers. In particular, HIS284, LYS287, and LYS291 are involved in most of the intermediate or transition states across all five paths. Furthermore, ASP464 interacts with tDNA at position t4 in the intermediate state for path 2 (Supplementary Figure S2); GLY507 and LYS676 interact with either the backbone or bases at positions t2 and t3 of tDNA in the intermediate states of path 3 (Supplementary Figure S3); ASN295 and SER283 interact with position t9 of tDNA in the intermediate state 1 and transition state of path 4, respectively (Supplementary Figure S4). All these results indicate that the polar interactions between tDNA and *Cb*Ago are critically involved in seed region recognition.

**Figure 3:**
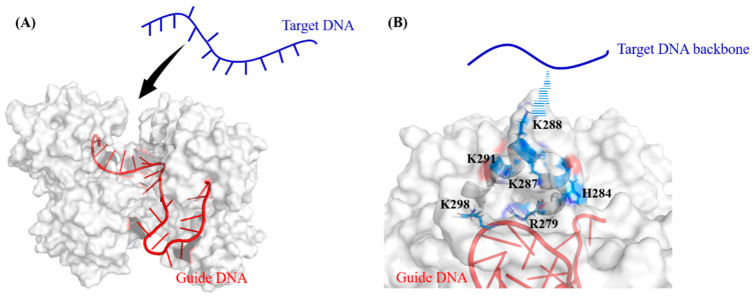
The process and mechanism of the *Cb*Ago–guide strand complex in recognizing tDNA in the seed region. (**A**) Schematic diagram of a free state tDNA moving towards the gDNA/*Cb*Ago complex. (**B**) Schematic diagram of the tDNA attracted by the positively charged α-helical secondary structure nearby the seed region of gDNA.

Although the free state tDNA fragment (Figure 3A) is allowed to move towards the *Cb*Ago protein from diverse orientations (initial structures), positively charged residues distributed in the structural domains of the nearby *Cb*Ago protein participate in capturing the negatively charged phosphate backbone of the target strand (Figure 3B). This structural domain adopts an α-helical secondary structure, with positively charged residues, including ARG279, HIS285, LYS287, LYS288, LYS291, and LYS298 (Figure 3B). However, these positively charged residues are involved in seed region recognition in two distinct ways. On one hand, once the target strand is captured and brought close to the pairing position in the seed region, it becomes difficult for the target strand to move away from the guide strand due to the attraction between the positively charged residues. On the other hand, if base mismatches exist between the target strand and the guide strand, such strong electrostatic attraction between the tDNA backbone and these positively charged residues may offset the free energy penalty of mismatched base pairing. This rationalizes the tolerance of mismatched base pairing in the seed region as observed in the current version of *Cb*Ago [1].

### 2.2. Simulation Study of a Charge-Attenuated CbAgo Mutant

**Figure 4:**
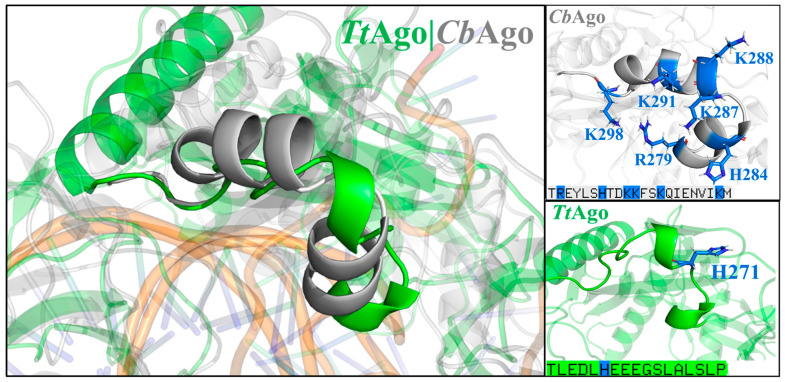
Structure alignment of the α-helical secondary structure domain in regions adjacent to the target strand of *Cb*Ago and *Tt*Ago. The individual structures and sequences of *Cb*Ago and *Tt*Ago are shown in the right panel. All positively charged residues are highlighted. *Cb*Ago is colored in gray and *Tt*Ago is colored in green.

We further analyzed and identified the key residues responsible for off-target effects in the seed region of *Cb*Ago by comparing the secondary structures and protein sequences of *Cb*Ago and *thermus thermophilus* argonaute (*Tt*Ago) [21,22] (Figure 4). Notably, *Tt*Ago does not tolerate a single mismatch in the seed region [21]. Unlike *Cb*Ago, *Tt*Ago possesses only one positively charged residue within the same α-helical secondary structural domain [23] (Figure 4). This suggests that molecular recognition in the seed region of *Tt*Ago is predominantly governed by base pairing. These findings reinforce our hypothesis that the positively charged residues in the structural domain of *Cb*Ago are primarily responsible for its tolerance of a single mismatch in the seed region without compromising its cleavage function.

Building upon these findings, we designed a mutant, m*Cb*Ago, in which all positively charged residues in the α-helical domain were mutated to alanine. Consequently, the recognition and pairing of a free target strand by m*Cb*Ago in the seed region are theoretically determined predominantly by base complementarity between the two strands. This alteration disallows mismatches between the duplexes and severely impedes the rate of seed region recognition and pairing when a mismatch occurs.

We then constructed five initial transition paths for m*Cb*Ago with the same 9-mer tDNA fragment (mpath 1–5), using the optimized paths of *Cb*Ago as templates. For each mpath, we performed random single base mutations at the g4 to g8 positions of the gDNA, resulting in ten additional initial transition paths for the mutated gDNA/m*Cb*Ago complex recognizing the 9-mer tDNA fragment (m1mpath 1–5 and m2mpath 1–5). Utilizing the same protocols as above, each of the 15 transition paths was re-optimized, and its free energy profile was re-estimated.

**Figure 5:**
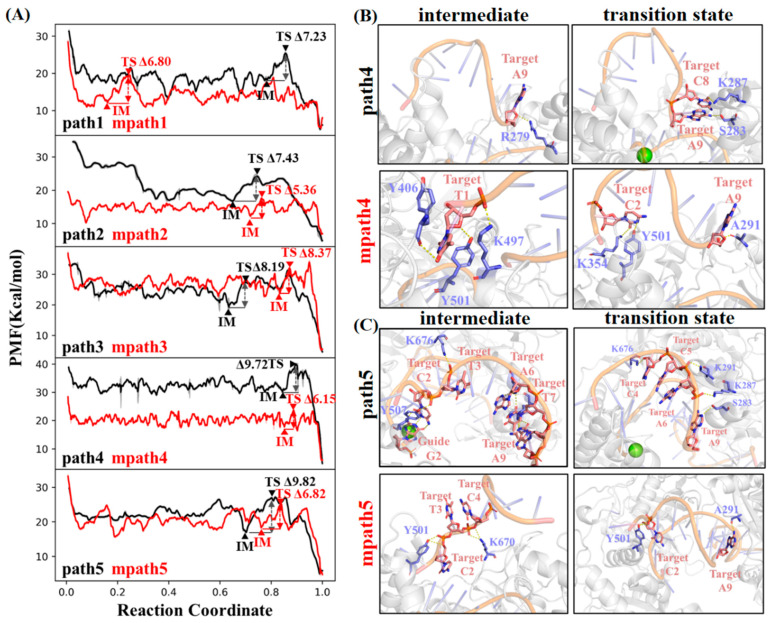
(**A**) Comparison of free energy profiles of the well-paired 9-mer tDNA fragment recognized by *Cb*Ago (path 1–5) and m*Cb*Ago (mpath 1–5). (**B**) The structure transitions dominating the dG_max_(IM→TS) of path 4 and mpath4, respectively. Polar residues involving the tDNA–protein interactions are labeled. (**C**) The structure transitions dominating the dG_max_(IM→TS) of path5 and mpath5, respectively. Polar residues involving the tDNA–protein interactions are labeled.

In comparison to *Cb*Ago (dGmax(IM→TS) ~7–10 kcal/mol, path 1–5, Figure 5A), the highest free energy barrier along the pathway for the well-paired 9-mer tDNA fragment binding to the seed region of the gDNA/m*Cb*Ago complex is significantly reduced (dGmax(IM→TS) ~5–8 kcal/mol, mpath 1–5, Figure 5A). This indicates that the removal of electrostatic interactions between the α-helical secondary structure of *Cb*Ago and tDNA effectively lowers the difficulty of base pairing.

A quantitative comparison of the differences between *Cb*Ago and m*Cb*Ago regarding dGmax(IM→TS) reveals that the decrease is most pronounced in mpath 4 and 5 (ddGmax(IM→TS) of −3.57 kcal/mol and −3.0 kcal/mol, respectively, Table 1). In mpath 4, the dGmax(IM→TS) is observed due to changes in tDNA–protein interactions from the t1 to the t2 and t9 positions of tDNA (Figure 5B). The tDNA–protein binding remains dominated by several polar residues, such as LYS354, TYR406, LYS497, and TYR501, but no polar residues from the α-helical domain (e.g., ARG279, SER283, and LYS287) are observed in path 4 (Figure 5B). Similarly, the dGmax(IM→TS) of mpath 5 is attributed to changes in tDNA–protein interactions from the t2, t3, and t4 positions to the t2 and t9 positions of tDNA (Figure 5C). Three positively charged residues, TYR501, LYS670, and LYS676, are involved in this transition, with none located in the α-helical domain. These results indicate that in m*Cb*Ago, dGmax(IM→TS) is no longer dominated by tDNA–protein interactions within the α-helical domain.

**Figure 6:**
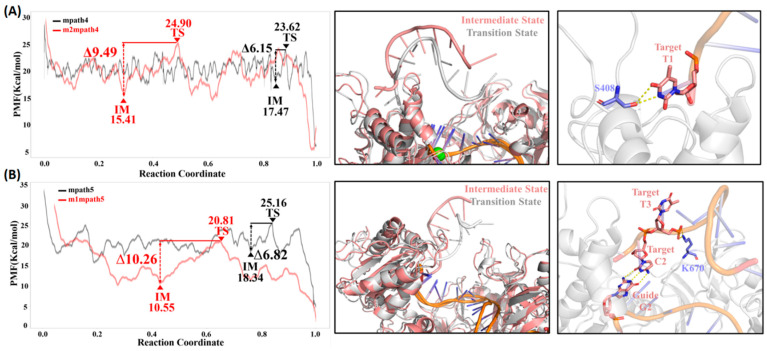
(**A**) Free energy profile of the well-paired and mismatched tDNA fragments recognized by m*Cb*Ago (mpath 4 and m2mpath4). Structural alignment of the intermediate and transition states dominating the dG_max_(IM→TS) of m2mpath4 is shown in the middle panel, and the detailed tDNA–protein interaction at the transition state is shown in the right panel. (**B**) Free energy profile of the well-paired and mismatched tDNA fragments recognized by m*Cb*Ago (mpath 5 and m1mpath5). Structural alignment of the intermediate and transition states dominating the dG_max_(IM→TS) of m1mpath5 is shown in the middle panel, and the detailed tDNA–protein interaction at the transition state is shown in the right panel.

Finally, we characterized the binding specificity of tDNA to the gDNA/m*Cb*Ago complex by examining the difference in dGmax(IM→TS) between mismatched and well-paired gDNA (ddGmax(IM→TS), Table 1). Consistent with our expectations, the free energy barriers encountered during the binding of the tDNA fragment to mismatched gDNA/m*Cb*Ago are consistently higher than those for well-paired sequences (ddGmax(IM→TS) is always positive, Table 1), indicating an increased difficulty in achieving seed region recognition and binding. The largest ddGmax(IM→TS) observed is approximately 3 kcal/mol, noted in both m2mpath4 and m1mpath5 (Figure 6). Indeed, we found that no base pairing occurs between the t3 to t9 positions of tDNA and the mismatched gDNA during the transition process of dGmax(IM→TS) in these two pathways (Figure 6). These results provide direct evidence of enhanced sequence specificity in the recognition of tDNA by m*Cb*Ago.

## 3. Materials and Methods

**Simulation parameters:** GROMACS 2019.4 [24] was used as the MD simulation platform, with the Amber14sb [25] and OL15 force fields [26] coupled with the TIP3P [27] water model to characterize the atomic-level interactions between *Cb*Ago and DNA. Sodium (Na^+^) and chloride (Cl^−^) ions were incorporated into the system to ensure electrostatic neutrality. Periodic boundary conditions were applied to all simulation boxes. Approximately 50,000 water molecules were added to a cubic box with dimensions of 120 Å. All systems were energy-minimized using the conjugate gradient method. Energy minimization was carried out for 10,000 steps using the steepest descent algorithm, followed by the conjugate gradient algorithm. Subsequently, a 100 ps NVT simulation was performed at 310 K for solvent equilibration, followed by a 1 ns NPT equilibration to 1 atm using the Berendsen barostat [28]. The production MD runs were conducted in an isothermal–isobaric ensemble using the Bussi (V-rescaling) [29] thermostat at 310 K and the Parrinello–Rahman barostat [30] at 1 bar. Long-range electrostatic interactions were handled by the Particle Mesh Ewald method [31], with a short-range cutoff distance of 10 Å for electrostatics and van der Waals interactions. A time step of 2 fs was chosen. Bonds were constrained using the LINCS [32] algorithm. Trajectories were recorded every 2 ps. A total of 200 ns runs were conducted. Each independent run started from the same initial structure but with different initial velocities randomly drawn from a Maxwell–Boltzmann distribution.

**Path optimization**: For the initial paths by SMD, the box building and equilibrium settings were the same with the MD simulation mentioned in simulation parameters. The umbrella pulling method (harmonic potential) was used for non-equilibrium pulling, with the spring constant for the harmonic potential set to 5000 kJ/mol/nm^2^. The pulling was performed along the direction of the vector containing the two pulling groups, with the atoms of target DNA and guide DNA defined as group1 and group2, respectively. For constant-velocity pulling, the displacement rate along the pulling coordinate was set to 0.01 nm/ps. The dimensions (X, Y, Z) along which the pulling force was applied were set for 8 paths as follows: ‘NNN’, ‘NNY’, ‘NYN’, ‘YNN’, ‘YYN’, ‘NYY’, ‘YNY’, and ‘YYY’. Here, ‘Y’ and ‘N’ represent whether a pulling force is applied in the respective direction (N means no, Y means yes). Of the eight initial paths, three involved the target DNA not being pulled away from the guide DNA. The dimension settings for the five selected paths to be optimized were ‘NNY’, ‘NYN’, ‘YNN’, ‘YYN’, and ‘NYY’. All conformational transition paths were optimized by a traveling-salesman-based automated path searching method (TAPS) [17]. The path optimizations were conducted by the TAPS python script (https://github.com/liusong299/TAPS, accessed on 25 March 2025), employing GROMACS-2019.4 [24] and PLUMED-2.5.3 [33,34] as conformational sampling engines. The conformation sampling simulations were performed in the NVT ensemble at 310 K using the velocity–rescale thermostat. The initial paths were determined by selecting conformations with a gap of 1.4 Å from the SMD trajectories. A total of 10,000 ps of sampling was conducted in each TAPS iteration. Gaussians of height 2.0 kJ/mol and width 1.0 were deposited every 0.02 ps, with frames recorded at the same frequency. After optimization, convergence was assessed using the MDS method [20] and PCV-z [35] analysis.

**Free energy estimation:** Umbrella sampling [18] was conducted using the GROMACS-2019.4 and PLUMED-2.5.3 software packages. The free energy profiles of the MEFPs were calculated along the PCV-s (reaction coordinates), which represents the progress along the LFEP. The sampling in each window was restrained within 1.0 Å of LFEP through a harmonic wall potential with a force constant of 20,000 kJ/(mol·Å^4^) at PCV-z = 4 Å^2^. Root-mean-square deviation (RMSD) calculations were performed among conformations, considering all atoms of both DNA strands and the heavy atoms of the *Cb*Ago protein residues in close proximity to the target and guide DNA. Structural alignment was performed based on the backbone atoms of the *Cb*Ago protein. The window size was adopted as 0.25 Å along the LFEP. Within each window, a force constant of 200 kJ/mol was applied, and the simulations were run for a minimum duration of 2 ns. A complete free energy profile was subsequently derived via the weighted histogram analysis method (WHAM) [36]. Upon convergence of the WHAM iterative process, the mean free energy and its associated standard statistical error (represented by error bars) were computed for each window.

## 4. Conclusions

*Cb*Ago, as a programmable endonuclease that can cleavage DNA targets at mild temperatures, has not yet been successfully applied in genome editing, mainly due to its high off-target rate. To dissect the origin of such high off-target rate and, therefore, ultimately enhance its fidelity, we elucidated the recognition mechanism of a 9-mer target DNA fragment by the seed region of the guide sequence through five transformation pathways. Our findings revealed that the prominent distribution of positively charged residues on a α-helical domain nearby the DNA–protein interface (ARG279, HIS285, LYS287, LYS288, LYS291, LYS298) significantly facilitates the rapid binding of these residues to the DNA phosphate backbone. However, the excessive existence of positively charged residues also diminishes the importance of base complementarity during the base-pairing of the gDNA–tDNA duplexes. Their role in seed off-target recognition is further confirmed by additional mutant simulations. Our designed mutant with alanine substitution to these residues exhibited higher free energy barriers and, therefore, slower recognition of off-target sequences than on-target ones. Collectively, these results offer critical rationale for future fidelity enhancement of *Cb*Ago via the integration of physical insights, protein–language models and experimental validation.

## Figures and Tables

**Table 1:** The highest free energy barrier of the conformational transition of tDNA fragment from five dissociated states (#1–#5) to the state of lowest free energy. Here, dG and ddG are the aliases of dG_max_(IM→TS) and ddG_max_(IM→TS), respectively.

	dG	dG	ddG	dG	ddG	dG	ddG
	Path	Mpath	Mpath-Path	M1mpath	M1mpath-Mpath	M2mpath	M2mpath-Mpath
#1	7.23	6.8	−0.43	8.34	1.54	9.06	2.26
#2	7.43	5.36	−2.07	7.44	2.08	8.17	2.81
#3	8.19	8.37	0.18	10.31	1.94	9.48	1.11
#4	9.72	6.15	**−3.57**	7.65	1.5	9.49	**3.34**
#5	9.82	6.82	**−3.0**	10.26	**3.34**	8.4	1.58
mean	8.48	6.7	−1.78	8.8	2.08	8.92	2.22
Std.	1.23	1.11	1.62	1.40	0.75	0.61	0.9

## Data Availability

The data have not been uploaded to a publicly available repository, and the data that support the findings of this study are available upon reasonable request to the corresponding author, Zhu.

## Supplementary Materials

The following supporting information can be downloaded at: https://www.mdpi.com/article/10.3390/ijms26104738/s1.
