# Supplementary material for: Excessive Existence of Positively Charged Amino Acids Caused Off-Target Recognition in the Seed Region of Clostridium butyricum Argonaute
Source: Int J Mol Sci. 2025 May 15;26(10):4738. doi: 10.3390/ijms26104738 (PMC12111869; doi:10.3390/ijms26104738)
Supplement: Supplementary file 1 [file ijms-26-04738-s001.zip › ijms-3579760-supplementary.pdf]

Supplementary Materials

Excessive Existence of Positively Charged Amino Acids Caused Off-Target Recognition in the Seed Region of *Clostridium butyricum* Argonaute

Wenzhuo Ma †, Wenping Lyu † and Lizhe Zhu *

School of Medicine, Warshel Institute for Computational Biology, The Chinese University of Hong Kong—Shenzhen, Shenzhen 518172, China; wenzhuoma@link.cuhk.edu.cn (W.M.); lvwenping@cuhk.edu.cn (W.L.)

* Correspondence: zhulizhe@cuhk.edu.cn

† These authors contributed equally to this work.

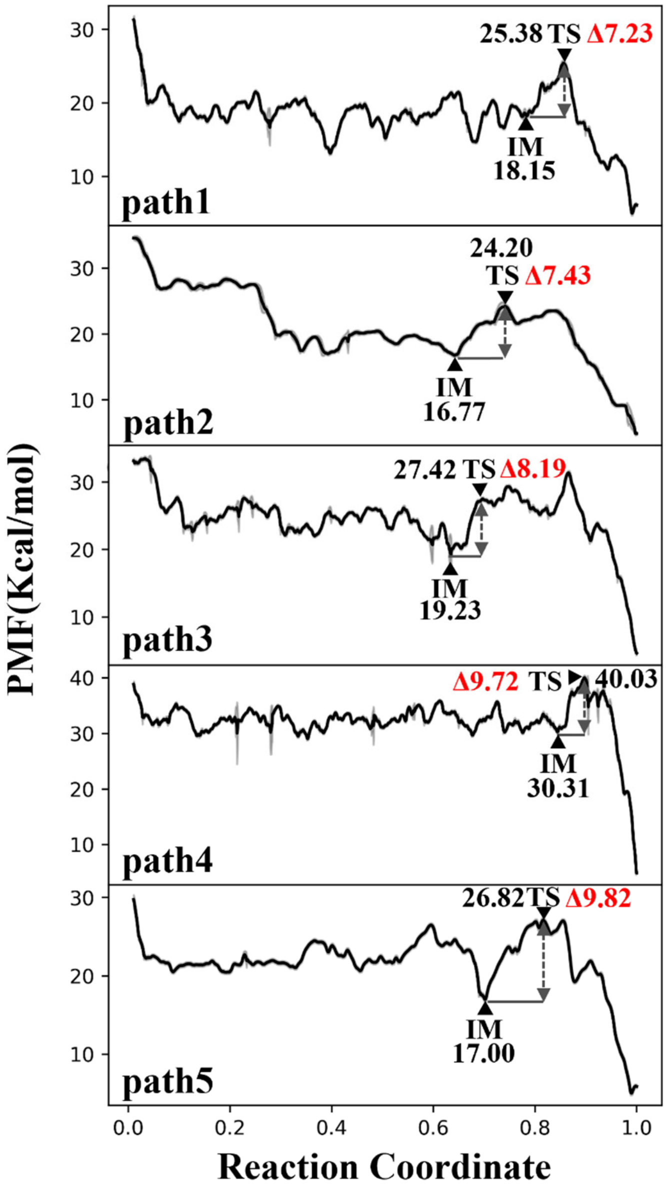

Figure S1. free energy profile of path 1-5. The highest free energy barrier between adjacent IM and TS (dGmax(IM→TS)) on each path is labeled.

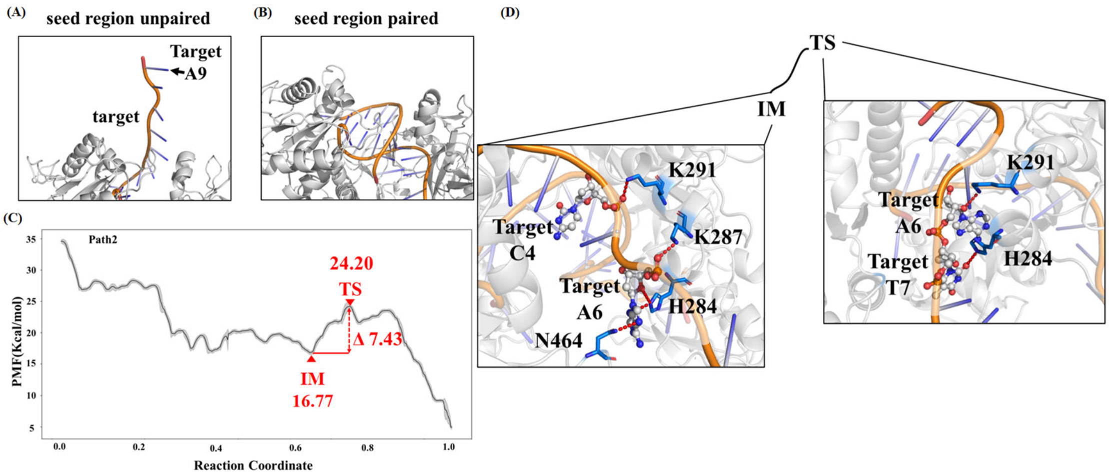

Figure S2. (**A,B**) The initial and final states of path 2. (**C**) The free energy profile of conformational transitions along the path 2. (**D**) The protein-DNA interactions at intermediate (IM) and transition state (TS).

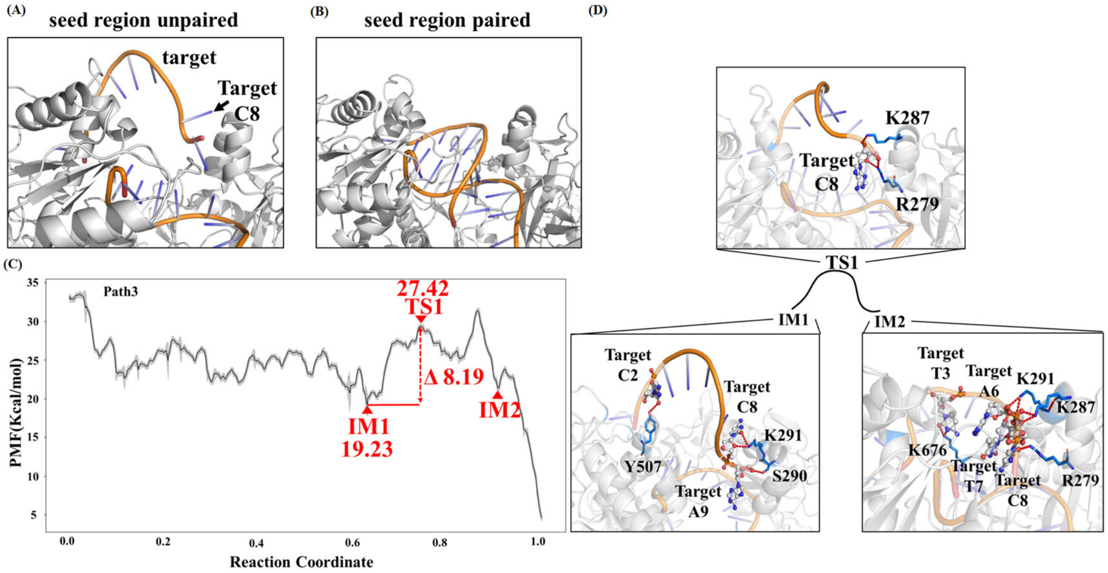

Figure S3. (**A,B**) The initial and final states of path 3. (**C**) The free energy profile of conformational transitions along the path 3. (**D**) The protein-DNA interactions at intermediates (IM1, IM2) and transition state 1 (TS1).

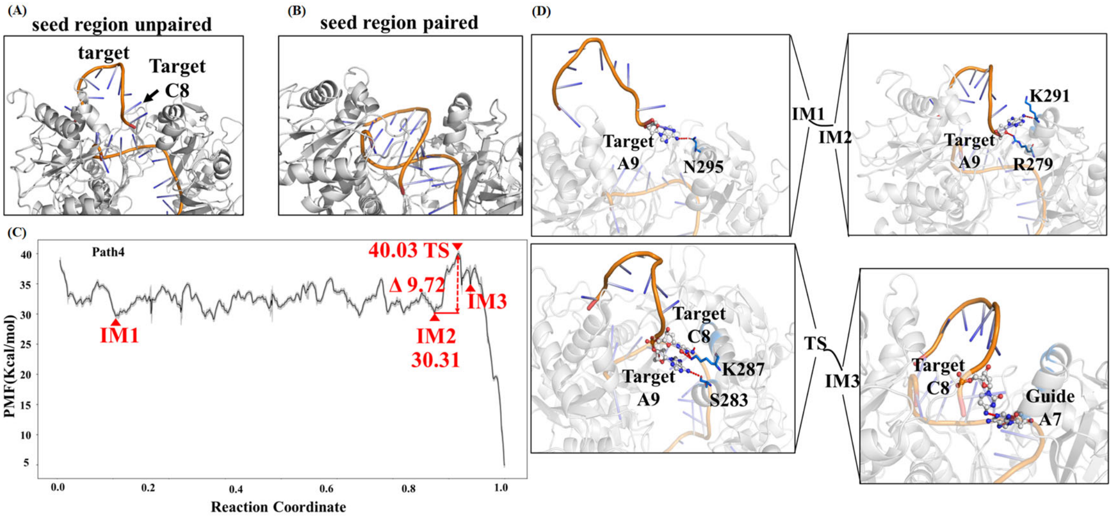

Figure S4. (**A**,**B**) The initial and final states of path 4. (**C**) The free energy profile of conformational transitions along the path 4. (**D**) The protein-DNA interactions at intermediates (IM1, IM2, IM3) and transition state (TS).

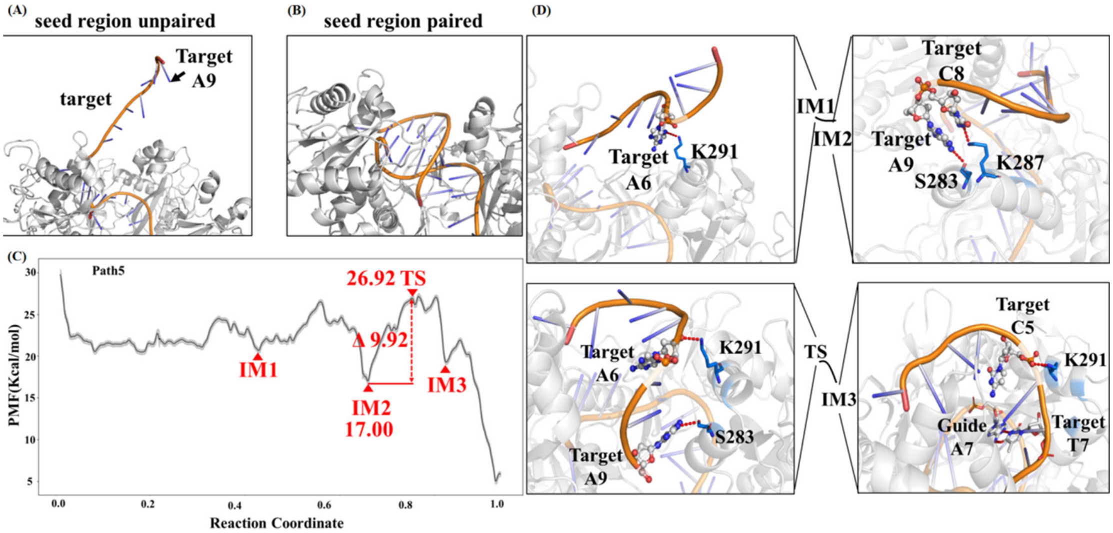

Figure S5. (**A**,**B**) The initial and final states of path 5. (**C**) The free energy profile of conformational transitions along the path 5. (**D**) The protein-DNA interactions at intermediates (IM1, IM2, IM3) and transition state 2 (TS2).
